# Translation of nanotechnology-based implants for orthopedic applications: current barriers and future perspective

**DOI:** 10.3389/fbioe.2023.1206806

**Published:** 2023-08-22

**Authors:** Long Chen, Chao Zhou, Chanyi Jiang, Xiaogang Huang, Zunyong Liu, Hengjian Zhang, Wenqing Liang, Jiayi Zhao

**Affiliations:** ^1^ Department of Orthopedics, Affiliated Hospital of Shaoxing University, Shaoxing, Zhejiang, China; ^2^ Department of Orthopedics, Zhoushan Guanghua Hospital, Zhoushan, China; ^3^ Department of Pharmacy, Zhoushan Hospital of Traditional Chinese Medicine Affiliated to Zhejiang Chinese Medical University, Zhoushan, China; ^4^ Department of Orthopedics, Zhoushan Hospital of Traditional Chinese Medicine Affiliated to Zhejiang Chinese Medical University, Zhoushan, Zhejiang, China

**Keywords:** nanotechnology, orthopedic, biomaterials, orthopedic implant, bioimplant

## Abstract

The objective of bioimplant engineering is to develop biologically compatible materials for restoring, preserving, or altering damaged tissues and/or organ functions. The variety of substances used for orthopedic implant applications has been substantially influenced by modern material technology. Therefore, nanomaterials can mimic the surface properties of normal tissues, including surface chemistry, topography, energy, and wettability. Moreover, the new characteristics of nanomaterials promote their application in sustaining the progression of many tissues. The current review establishes a basis for nanotechnology-driven biomaterials by demonstrating the fundamental design problems that influence the success or failure of an orthopedic graft, cell adhesion, proliferation, antimicrobial/antibacterial activity, and differentiation. In this context, extensive research has been conducted on the nano-functionalization of biomaterial surfaces to enhance cell adhesion, differentiation, propagation, and implant population with potent antimicrobial activity. The possible nanomaterials applications (in terms of a functional nanocoating or a nanostructured surface) may resolve a variety of issues (such as bacterial adhesion and corrosion) associated with conventional metallic or non-metallic grafts, primarily for optimizing implant procedures. Future developments in orthopedic biomaterials, such as smart biomaterials, porous structures, and 3D implants, show promise for achieving the necessary characteristics and shape of a stimuli-responsive implant. Ultimately, the major barriers to the commercialization of nanotechnology-derived biomaterials are addressed to help overcome the limitations of current orthopedic biomaterials in terms of critical fundamental factors including cost of therapy, quality, pain relief, and implant life. Despite the recent success of nanotechnology, there are significant hurdles that must be overcome before nanomedicine may be applied to orthopedics. The objective of this review was to provide a thorough examination of recent advancements, their commercialization prospects, as well as the challenges and potential perspectives associated with them. This review aims to assist healthcare providers and researchers in extracting relevant data to develop translational research within the field. In addition, it will assist the readers in comprehending the scope and gaps of nanomedicine’s applicability in the orthopedics field.

## 1 Introduction

Nanotechnology is a multidisciplinary field that manipulates the nanometer-scale chemical, physical, and biological properties and structures of materials. Nanomaterials have size-dependent characteristics that are not typically observed in large quantities of materials. The advancements in nanotechnology have opened up new possibilities for diverse applications in the fields of medicine (Kaur et al., 2015; Rani et al., 2015; Liu et al., 2019), molecular biology (Ulijn and Jerala, 2018; Kumar et al., 2020; Kumar et al., 2019a), biotechnology (Kumar et al., 2017; Rani et al., 2018), as well as environmental science (Bhanjana et al., 2019; Kumar V. et al., 2019; Kumar et al., 2019b; Castiglioni et al., 2017; Samanta et al., 2019; Gao et al., 2023). With the development of numerous modern techniques for the diagnosis, prevention, and treatment of multiple diseases, such as cancer treatment, drug delivery, medical imaging, scaffolds for tissue engineering, and immunotherapy, the use of nanotechnology in medicine (for instance, nanomedicine) has become more apparent.

Nanomaterials are viable means for the development of future orthopedic implants (Wang et al., 2016; Cheng et al., 2018) because of their capacity to imitate or recapitulate bone structure. In orthopedic applications, bone substitutes are necessary for treating irreversible damage to natural and healthy bone. Nanomaterials are expected to have a significant impact in this case, not only by providing structural support to cells (referred to as nanofunctionalized scaffolding), but also by influencing cell propagation, migration, and differentiation (Patel et al., 2016; Ferraris et al., 2016; Vieira et al., 2017; Antoniac et al., 2022; Feltz et al., 2022; Li et al., 2022).

Bone and its components, for example, Haversian systems, hydroxyapatite (HA), and collagen fibrils, are nano-compounds. Therefore, orthopedics is a fascinating nanotechnology application field. Orthopedic procedures commonly involve intricate interactions at the microscopic level between the host tissue and nanomaterials. Enhancing the efficiency of connections can be achieved by employing biomaterials comprising nanoparticles and structures to modify nanoscale materials. The statement mentioned above forms the foundation for a considerable portion of nanotechnology applications within the medical field of orthopedics. The application of nanotechnology in the field of orthopedic research demonstrates significant promise due to its ability to enhance the mechanical characteristics and biocompatibility of implantable orthopedic devices. Nanostructured grafts and prostheses offer several advantages, including enhanced mechanical durability, improved resistance to wear and corrosion, the potential for drug delivery, and the ability to serve as scaffolds for tissue regeneration. The two main classifications of biological tissues are hard tissues, which include teeth, cartilage, nails, and bone, and soft tissues, which encompass the epidermis, fibrous tissues, ligaments, and synovial membranes. These tissues may or may not contain mineral elements. The scarcity of organ donors prompted scientists to develop innovative techniques to mimic or replicate organs. Bioimplants have been specifically engineered to address the need for restoring, maintaining, or enhancing the functionalities of human tissues. Nevertheless, biomaterials designed for implants exhibit distinct characteristics compared to those found in natural tissues and bones. Biomaterials are synthetic or naturally occurring compounds developed for use in biological systems.

**FIGURE 1:**
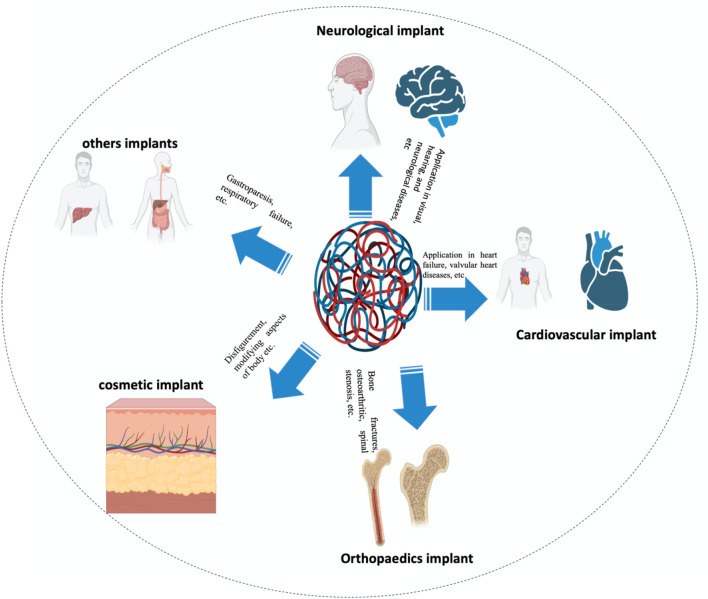
According to their application, bioimplants are categorized as neurological/sensory, cardiac, cosmetic, and orthopedic implants, among others.

The bioimplant market is expanding at an exponential rate as a result of an aging population, lifestyle changes (particularly those that induce and prolong chronic diseases such as osteoarthritis and cardiac disorders), bioengineering technological advancements, and increased cosmetic implant awareness. According to market research, the global bioimplant market is expected to reach $115.8 billion by 2020, expanding at a compound annual growth rate (CAGR) of 10.3% over the period projected (2014–2020) (Jackson, 2016). Bioimplants are now recognized as a potential treatment option for neurological diseases, vision problems, cardiac disease, orthopedic problems, deformities, and dental issues (Figure 1) (Ott et al., 2008; Ghezzi et al., 2013; Song et al., 2013; Jackson, 2016; Scaini and Ballerini, 2018). Various bioimplants, such as replacement of joints, vascular grafts, sutures, bone plates, heart valves, implants for teeth, ligaments, intraocular lenses (IOLs), and others, are typically used to (i) re-establish or restore the function of deteriorated or destroyed tissues, (ii) alter the function of a body component, (iii) aid in curing, and (iv) cosmetically repair abnormalities (Cross et al., 2016). Using conventional metallic/nonmetallic materials, several engineering techniques have been observed to mimic the physical properties, chemical attributes, and gradient architecture of tissues or organs. Conventional bioimplants do not always conform to the tissues, are not always compatible with the tissues, and may not be tolerated by the human body (Hobson, 2009; Huebsch and Mooney, 2009; Morigi et al., 2012; Bian et al., 2016; Rambaran and Schirhagl, 2022).

The impact of nanotechnology on the field of grafting has grown significantly in recent years. Researchers are encouraged to investigate the function of nanoparticles (NPs) in enhancing the performance of conventional grafts due to their biologically-inspired properties. This study investigated the evolution of orthopedic biomaterials from conventional (non-metallic and metallic) components to nanomaterials. In addition, this article discusses a variety of surface modification techniques, including surface structuring and nanotechnology-based therapies. This evaluation also focuses on antibacterial surface therapeutic interventions for bioimplants. The identification of treatment sites and their successful implantation are the primary focuses of orthopedic treatments. Despite the recent success of nanotechnology, numerous obstacles remain in the way of nanomedicine’s implementation in orthopedics. The purpose of this review is to offer a comprehensive examination of recent advancements, their commercial viability, obstacles encountered, and potential future opportunities. This analysis will assist healthcare professionals and researchers in synthesizing this information to design translational research in the field. Furthermore, this will support the readers in understanding the extent of applicability and constraints of nanomedicine in the field of orthopedics.

## 2 Biomaterials in orthopedic devices

Since ancient times, humans have used a variety of foreign substances for medical purposes, such as sutures made of hair or cellulose, as well as horns or wood for bone fracture repair (Griffin et al., 2004; Achterberg et al., 2014; Love, 2017; Ghasemi-Mobarakeh et al., 2019; Hacker et al., 2019; Heimann, 2020; Marin et al., 2020). Metals and alloys are the material of choice for implants in the modern era due to their superior strength, reduced risk of rejection, and inertness. Since the end of the second World War, there has been rapid and extensive development in the field of polymers, a novel chemical class that has been synthesized and subsequently utilized on a large scale (Mir et al., 2018; Donnaloja et al., 2020; Xu and Song, 2020). Implantable devices, irrespective of the material employed, are required to meet both general and specialized criteria, encompassing mechanical and biological properties.

### 2.1 Common material needs for orthopedic implants

The most essential criteria for implant materials are their clinical and production qualities. Regarding therapeutic requirements, implant materials must not be rejected by the body or produce hazardous byproducts (Tian et al., 2019). As a manufacturing criterion, materials must permit the production of the optimal design at a reasonable rate.

### 2.2 Material needs

The mechanical requirements of an implant are determined by its intended use, such as its loading strength and shear stress flexibility. In orthopedics, graft materials must withstand multiple upload/download cycles under torsional, bending, and shear stresses. In addition, implantable devices are frequently exposed to corrosive environments, which can modify their properties. Therefore, an accurate evaluation of mechanical characteristics is necessary to maintain fracture reductions. The material’s mechanical properties are measured in terms of the distortion (strain) brought on by an applied force (stress) (Pal and Pal, 2014). The application of stress can be induced by various mechanical actions, including loading, compression, torsion, bending, or shear. By utilizing stress-strain diagrams, which depict the deformation of a material when subjected to an external force (Silver and Shah, 2016), the mechanical characteristics of a graft substance may be evaluated. The diagram represents two distinct areas: in the former, deformation is permanent, whereas in the latter it is not. The slope of the curve in the elastic region indicates intrinsic rigidity or hardness (also known as Young’s modulus). Rigidity is the resistance to the deformation of a substantial body. As strain increases, micro-fractures form, plastic distortions appear, and eventually the substance breaks (Li et al., 2019). In contrast to implanted materials, bone rigidity is not continuous. Anisotropic is a term used to describe bone tissue. The mechanical properties of materials vary depending on the direction in which they are measured, which is referred to as anisotropy. Therefore, the mechanical characteristics of bones are dependent upon the direction in which force is applied. For example, the rigidity of bone tissue is greater when an external force is applied along the longitudinal axis of the bone, as opposed to being exerted on its surface (Bankoff, 2012).

The primary stress experienced in a pelvic fracture is the loading stress. The load-deformation curve can be obtained by deriving it from the general load curve, as demonstrated in prior research (Basso, 2015). The transformed diagram enables the determination of the implantation device’s rigidity in relation to that of a normal bone. In the case of a simulation, evaluating the rigidity of synthetic bones that mimic specific bone diseases, such as osteoporosis, is also advantageous. In the plastic zone, it is possible to determine the ultimate stress that causes a material to fracture. Consequently, rigidity and failure load are widely employed characteristics for defining a mechanical implant material (Basso, 2015; Li et al., 2019). Using load-deformation diagrams, it is possible to calculate the mechanical properties and resistance of an implant.

#### 2.2.1 Biological needs

**FIGURE 2:**
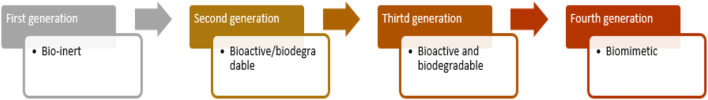
Categories of biomaterials.

The most important quality of an orthopedic device is its biological inertness or lack of response to its surrounding biological environment. Several reactions occur at the material surface in physiological circumstances; consequently, implant degradation is possible. This is permitted if the process does not compromise the mechanical strength or produce hazardous residues (Todros et al., 2021). In contrast to common belief, current research indicates that the acceleration of the restoration process can be achieved through the implementation of a carefully controlled reactivity between the implanted substances and the surrounding biological environment (Thanigaivel et al., 2022). The progress in biomaterials has facilitated the development of substances possessing unique characteristics that contribute to the compatibility of grafts with the morphological and mechanical properties of the recipient’s structure. According to Dolcimascolo et al. (Dolcimascolo et al., 2019), biomaterials used over the past 60 years can be categorized into three generations: bio-inert constituents (first generation), bioactive or degradable substances (second generation), and the current generation (third generation). The material implanted in the third generation is designed to stimulate molecular responses that accelerate the healing process. Recent research has led to the development of the fourth generation of biomaterials (Allo et al., 2012; Festas et al., 2020; Bhaskar and Nagarjuna, 2021; Basu et al., 2022). The classifications of biomaterials are shown in Figure 2.

This categorization depicts the theoretical development of implant material requirements. The primary requirements for the first generation were the rigidity and biological inertness of the material. The process of implantation results in the occurrence of non-specific protein absorption on the surface of the material. This leads to the formation of a fibrous tissue capsule, which encloses the graft. This encapsulation poses a risk to the implant (Gautam et al., 2022). Thus, the second generation of substances was developed. During the period from 1980 to 2000, the primary requirement for the second generation of implants was the advancement of bioactive and bioabsorbable materials, while simultaneously preserving the mechanical characteristics of the implants (Sheikh et al., 2015; Saad et al., 2018). A bioabsorbable material can degrade slowly, allowing for tissue regeneration and repair. Instead of a fibrous capsule, the bioactive substance is intended to cover its surface with HA, a naturally occurring component of bone. *In vivo* stimulation of the production of a hydroxyapatite layer on the implant surface is believed to enhance the mineralization, fixation, and bone regeneration processes (Zakaria et al., 2013; Arifin et al., 2014). The chemical bonding of various reactive groups to the surface of the polymer confers biofunctionality. Thus, this improvement was made possible by modifying the implant surface to promote specific cellular responses as opposed to nonspecific reactions as in the case of the first generation (Saad et al., 2018). The ability to trigger specific biological reactions at the molecular level is essential to the development of the third generation of biomaterials. These biomaterials apply to regenerative medicine, tissue transplantation, tissue engineering, and implantation. Certain biomaterials are intended to be temporary porous three-dimensional structures that stimulate tissue regeneration, nutrient transport, and potentially angiogenesis (Serrano and Ameer, 2012; Ning et al., 2016). The fourth generation of biomaterials creates a customized connection with microenvironments and biological processes by meeting fourth requirement: inertia, receptivity, activity, and autonomy (Montoya et al., 2021).

### 2.3 Future perspectives for the materials utilized in orthopedics

**FIGURE 3:**
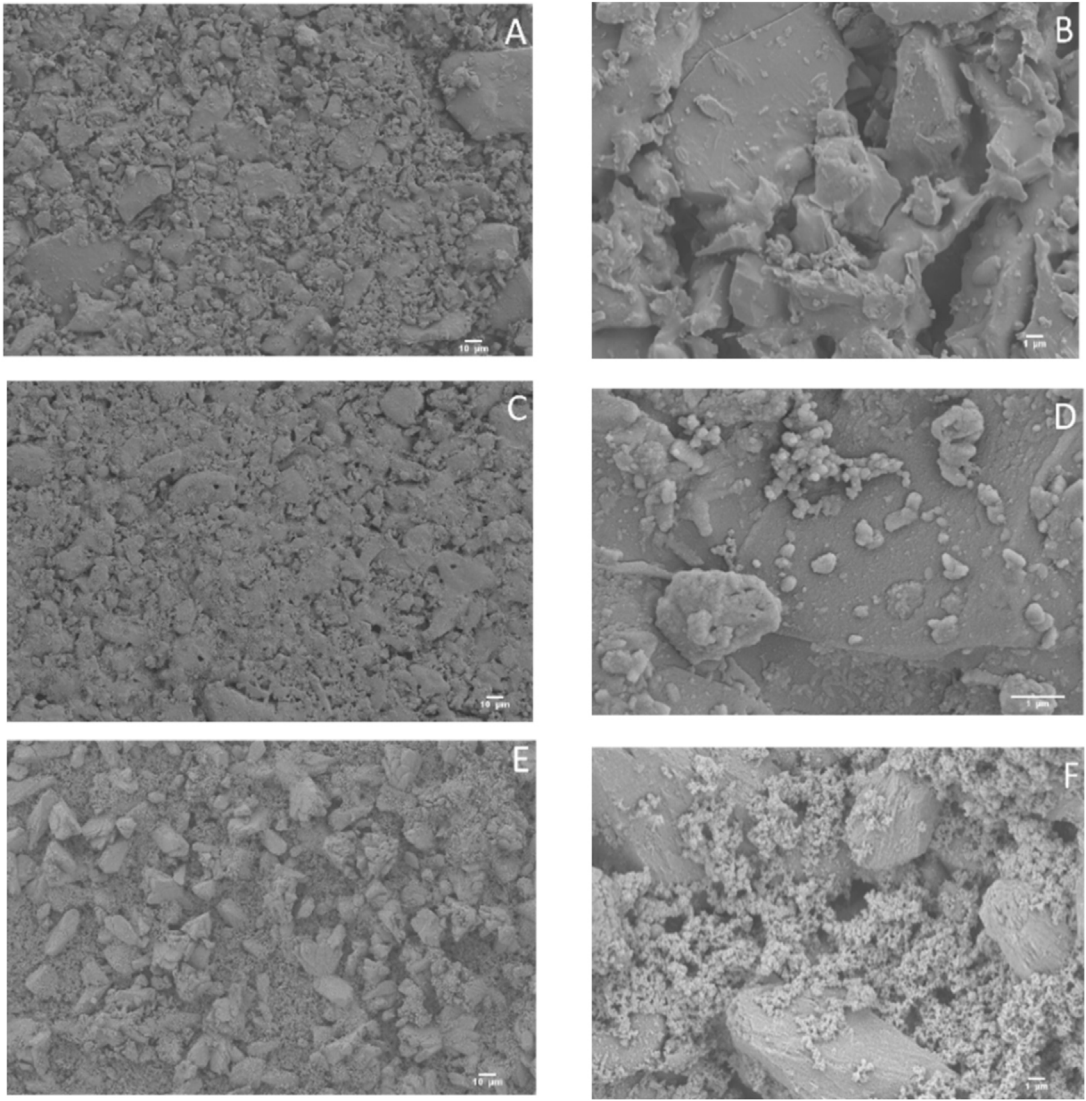
Scanning electron microscopy (SEM) micrographs of uncoated BGC1 **(A, B)**, BGC1@PDA **(C, D)**, and BGC1@PDA@Ag **(E, F)** (Tejido-Rastrilla et al., 2019).

Metal, polymers, and bioceramics are currently the most commonly used materials in orthopedics (Filip et al., 2019a; Badulescu et al., 2019; Filip et al., 2019b; Badulescu et al., 2020; Badulescu et al., 2021; Ma et al., 2021; Pattanayak and Sahoo, 2021; Tsakiris et al., 2021). Recent data indicate that biomedical nanotechnology is receiving increased attention (Soni et al., 2018; Kalyanaraman et al., 2019; Li et al., 2020; Nikolova and Chavali, 2020). Ceria nanoparticles or nano-ceria (CeO_2_-NPs) have greater potential applicability in orthopedics, according to research (Luo et al., 2023). Surface zeta potentials of the bioactive glass-ceramic BGC1 and the functionalized samples BGC1@PDA@Ag and BGC1@PDA were all significantly negative, as desired for *in vitro* biocompatibility. BGC1 and BGC1@PDA were found to be non-toxic using the MG-63 cell line. The biocompatibility of BGC1@PDA@Ag was moderate. According to antibacterial tests, BGC1@PDA@Ag exerts a potent antimicrobial effect on both Gram-negative and Gram-positive bacterial strains. The current experimental findings did not reveal any observable antibacterial properties of PDA. The inherent attributes of silver-incorporated bioactive glasses exhibit considerable potential for the utilization of nanotechnology in the field of orthopedics (Tejido-Rastrilla et al., 2019) (Figure 3).

**FIGURE 4:**
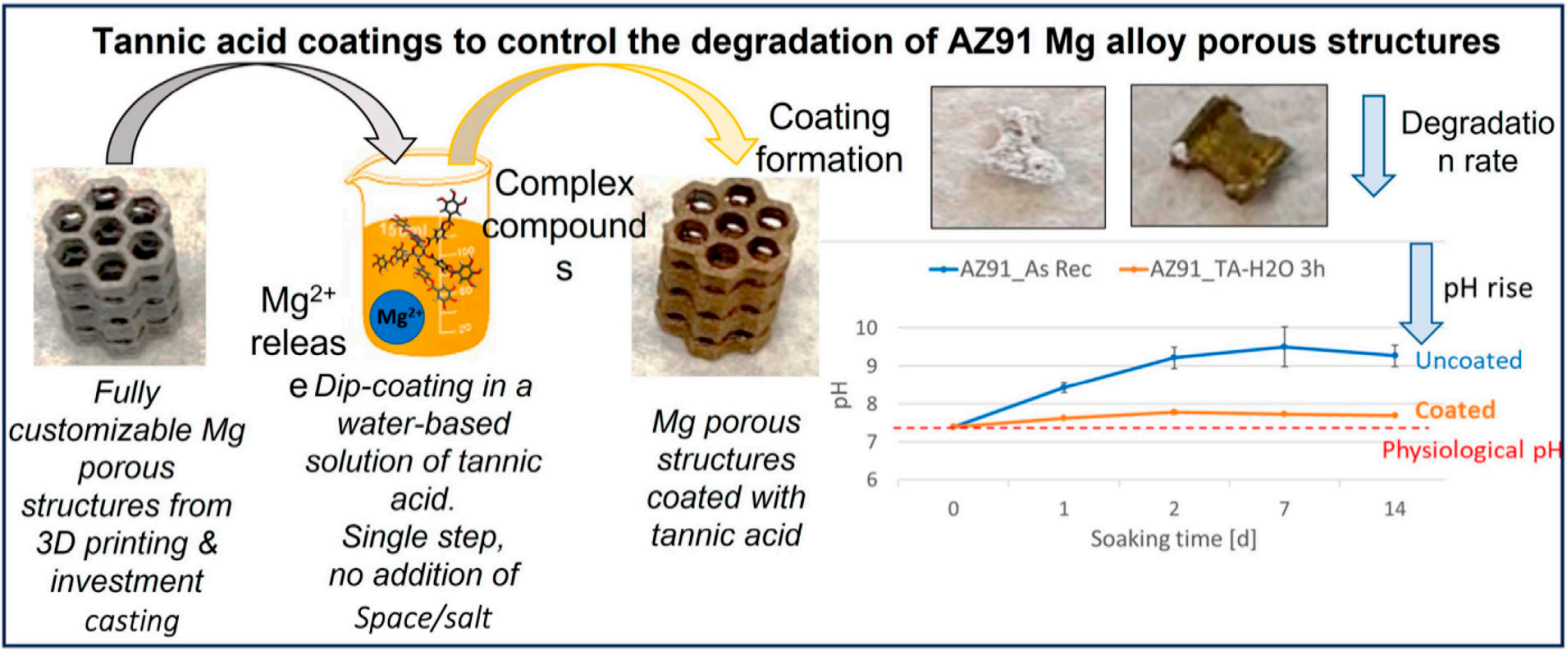
Tannic acid coatings to manage the breakdown of AZ91 Mg alloy porous structures (Spriano et al., 2023).

Magnesium alloys have now become the object of intense research due to their biocompatibility and acceptable mechanical qualities. Holweg et al. (2022), investigated uniform deterioration in intraoperative clinical locations of human bone supported with magnesium screws and a satisfactory interface between bone and implant. The application of the coating was performed on substrates that had not undergone any treatment, except for rinsing. The aforementioned technique exhibits superior efficiency in terms of speed (requiring only a single passage without the need for substrate pretreatment), durability (eliminating the use of harmful substances), and cost-effectiveness (reducing both process time and reagent consumption) compared to the methods proposed in existing literature (Spriano et al., 2023) (Figure 4). Recent research has emphasized the development of innovative substances and surface modification techniques that resemble human bone more closely. 3D printing technology is becoming increasingly essential in orthopedics; however, orthopedic grafts have not been thoroughly investigated. Polymers are frequently used as filaments, stereolithography equipment solutions, and direct ink-writing mediums in 3D-printed bone replacements (Memarian et al., 2022).

## 3 Nanoengineered biomaterials for orthopedic tissue interface regeneration and repair

Nanoengineered biomaterials and nanofabrication methods have emerged as viable alternatives to conventional techniques for simulating biological tissues (Park et al., 2007; Gaharwar et al., 2014a; Carrow and Gaharwar, 2015; Kerativitayanan et al., 2015). Due to enhanced control over the mechanical, structural, and chemical properties of nanoengineered substances, cells transplanted on or within these 3D scaffolds can support and simulate specific biological characteristics of natural tissue contacts. Various nanofabrication techniques, such as phase separation and electrospinning, may modulate the biological complexity and scaffold spatial geometry (Tuzlakoglu et al., 2005; Zhang and Webster, 2009; Zhao et al., 2013; Gaharwar et al., 2014b). The manipulation of drug release by nanoscale scaffolds can exert a regulatory influence on cellular activity. To reproduce certain biological structures, it is possible to fabricate intricate geometries such as fibers, sheets, spheres, networks, and hollow tubes. This review is limited to nanomaterials with dimensions less than 500 nm. Specifically, we will conduct a critical evaluation of the various nanomaterials currently utilized in orthopedic interface restoration.

Several polymeric and ceramic nanomaterials have been used to engineer orthopedic tissues including cartilage, bone, ligaments, and tendons (Zhang and Webster, 2009; Castro et al., 2012; Kerativitayanan et al., 2015). Due to their high bioactivity, ceramic nanomaterials such as calcium phosphate, hydroxyapatite, bioactive glasses, and nanosilicates have been utilized to treat rigid tissues, such as bone (Gaharwar et al., 2014a; Kerativitayanan et al., 2015). Hydroxyapatite (HAp), a nanoparticle extensively investigated in the context of orthopedic grafts (Liu et al., 2009; Zandi et al., 2010; Ravichandran et al., 2012), is the prevailing subject of study in relation to bone regeneration. Due to its resemblance to the biological apatite found in bone tissue, HAp demonstrates exceptional potential as a biomaterial for bone regeneration. Additional bioactive materials comprise bioactive glasses, calcium phosphate, and silicates. Osteogenic differentiation can be triggered by silicate NPs, which are 2D NPs (Gaharwar et al., 2013; Chimene et al., 2015). The incorporation of nanosilicates into hydrogels resulted in enhanced mechanical properties, rendering them suitable for utilization in bone scaffolds (Gaharwar et al., 2011; Gaharwar et al., 2012; Xavier et al., 2015). Nanosilicates are increasingly being recognized as a promising material for bone repair, although with a lesser extent of research compared to nHAp. The ceramic nanoparticles exhibit complex mineral structures that have been proven to effectively adhere to neighboring bone tissue and stimulate the process of bone regeneration. In recent years, several carbon-based nanostructures, such as graphene, nanodiamonds (NDs), and carbon nanotubes (CNTs), have been investigated for bone tissue engineering (TE) (Goenka et al., 2014). Graphene has been found to stimulate stem cell osteogenic differentiation (Nayak et al., 2011), and graphene oxide demonstrates a comparable ability (Tatavarty et al., 2014). Few types of NPs have been investigated for the treatment of delicate orthopedic tissues such as tendons, cartilage, and ligaments. Nanosheets of titanium dioxide (TiO_2_) were investigated for cartilage tissue (Liu et al., 2015). The nanosheets were combined with an acrylamide hydrogel to produce a nanocomposite possessing physical and chemical properties comparable to those of natural articular cartilage. Nanofibers are commonly employed for ligament and tendon tissues due to their fibrous structure. Several polymeric biomaterials, such as poly (lactic-co-glycolic acid) (PLGA), collagen, poly (L-lactic acid) (PLLA), and poly (caprolactone) (PCL), have been employed in the fabrication of nanofibers (Zhang et al., 2005; Moffat et al., 2009; Xie et al., 2010; Liu et al., 2015). A number of the aforementioned nanomaterials have yet to be explored in the context of interface tissue engineering, with only a limited selection of nanomaterials having been specifically designed and studied for this application. Various nanofabrication techniques are utilized to obtain nanoengineered scaffolds composed of both natural and synthetic polymers, such as PLGA, PCL, PLLA, collagen, silk, hyaluronic acid, fibrin, and alginate. Typically, these biomaterials have undergone modifications for practical applications. In certain cases, they are combined with other polymers and NPs (such as calcium phosphate and hydroxyapatites) to enhance their mechanical and bioactive properties (Gaharwar et al., 2014a; Carrow and Gaharwar, 2015; Kerativitayanan et al., 2015). Specifically, the manipulation of nanoscale topographies through the incorporation of NPs into a polymeric framework has been shown to exert an influence on cellular outcomes (Sun et al., 2007). Due to the fact that nanocomposite materials can induce morphological changes, gene expression, cell proliferation, and differentiation, and mimic the composition of natural tissue (Pek et al., 2010), their use has increased over the past two decades.

## 4 Nanoscale methods for designing multilayer and gradient structures

**FIGURE 5:**
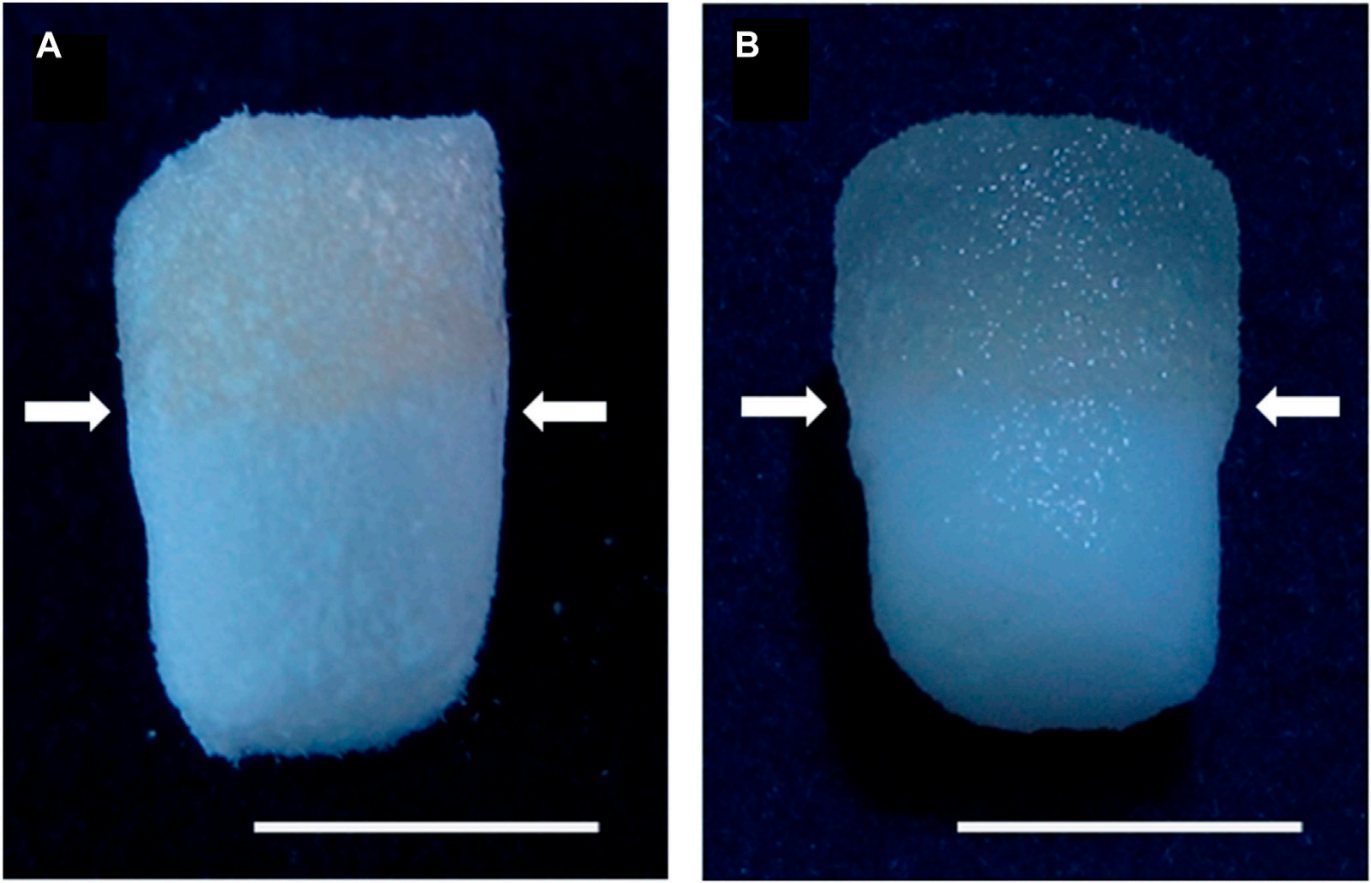
Both dried **(A)** and moist **(B)** biphasic scaffolds are composed of mineralized salmon collagen (the bottom layer) and fibrillized jellyfish collagen (the top layer). The transition zone between the strata is denoted by arrows. The scale bar represents 5 mm (Gelinsky et al., 2007).

Currently, numerous fabrication techniques are used to create orthopedic interface tissues. Growth factors and/or cells are utilized in monolithic scaffolds in the most fundamental technique (Figure 5) (Gelinsky et al., 2007). This method was commonly used to model a single tissue type, such as cartilage or bone, but it cannot represent multiple tissue types for interface tissues. Recent research has concentrated on bi-layered scaffolds, with each layer representing a distinct tissue type (Ghosh et al., 2008; Qu et al., 2011; Yan et al., 2015). While this particular strategy offers a more precise representation of the complex interface tissue, it fails to account for the interface area (Dormer et al., 2010). In recent times, the incorporation of three or more layers has become a prevalent aspect in the design of multilayered scaffolds. The middle layer(s) in this strategy represent the interface region, while the exterior layers imitate rigid or soft tissue (Liverani et al., 2012; Soo Kim et al., 2014; He et al., 2015). Various substances and cell types can be utilized in these layered designs to mimic the complex structures of interface tissues. However, it is important to note that the transition between the two depicted tissues may not always be smooth and continuous. The development of a gradient scaffold is considered to be a novel approach in the field of tissue engineering, aimed at replicating the properties of interface tissues (Singh et al., 2008; Dormer et al., 2010; Sant et al., 2010). In this method, the materials or chemical composition are modified progressively to more closely resemble transitional native tissues. The progressive transition can result in diverse cellular expression and the formation of a diverse environment. Several of the discussed methods employ the gradient method, as well as the creation of a chemical or material gradient using tilt angle, capillary action, microfluidics, or centrifugation (Oh et al., 2007; Sant et al., 2010).

## 5 Nanoengineered bone-cartilage interface

The primary objective of the TE interface is to restore, enhance, or restore bone-tissue interactions that have deteriorated. Cartilage lesions are typically difficult to treat because the injury can affect both the subchondral bone and the articulating cartilage, or the osteochondral junction in particular. The clinically relevant osteochondral methods for cartilage renewal include osteochondral and chondrocyte transplants, as well as debridement of injured tissues (via arthroscopy). Surgical procedures frequently require the removal of the damaged bone cartilage region by creating an osteochondral defect.

Microfracture is another common surgical treatment for comparable injuries, in which calcified cartilage is separated and the subchondral bone is perforated to create a defect. Through the creation of small openings, the defects are filled with bone marrow components containing stem cells. It is one of the most commonly used treatments for cartilage damage (Sridharan et al., 2016), despite frequently producing less durable and disorganized tissue. Unfortunately, a significant portion of these therapeutic techniques exhibits suboptimal efficacy, leading to the emergence of undesirable patient complications (Robinson et al., 2003; Wang et al., 2009). Current advancements in the field are focused on the development of less invasive techniques for the restoration of cartilage. These techniques involve the utilization of various polymeric scaffolds. For instance, second-generation Autologous chondrocyte implantation tissue engineering cartilage graft (Ossendorf et al., 2007), monolayer-expanded cartilage cell product with a hydrogel (Robinson et al., 2003; Selmi et al., 2008). Another approach is the implantation of a 3D collagen type I gel with autologous chondrocytes (Andereya et al., 2006), or the use of a tissue-engineered collagen matrix implanted with autologous chondrocytes (Crawford et al., 2009). Additionally, hydroxyapatite with interconnected pores (Adachi et al., 2007), as well as matrix-assisted autologous chondrocyte implantation (Panagopoulos et al., 2012) have been employed to facilitate the restoration of cartilage. In addition, a cell-free devitalized bovine trabecular bone cylinder, a bone replacement derived from the bovine origin, and Chondro-Gide have been asserted to assist in osteogenic recovery (Scotti et al., 2010). Though, bovine trabecular bone cylinder induces xenogenic responses, and because of a lack of clinical evidence, this product is not a clinician’s first option (Auyeung et al., 2010).

In addition to b-TCP, hydroxyapatite nanoparticles (nHAp) are commonly used in osteochondral and osteogenic restoration techniques. In a separate investigation (Liu et al., 2009), collagen scaffolds consisting of nHAp crystals were fabricated using a chemical reaction gradient involving calcium chloride and disodium hydrogen phosphate. However, it should be noted that this investigation did not include any *in vitro* confirmation of cellular responsiveness to the stepped scaffold. The investigation also encompassed the examination of agarose gels and alginate combined with nHAp as potential materials for the regeneration of the osteochondral interface (Khanarian et al., 2012). The use of alginate scaffolds did not yield consistent dispersion of nano and micro-sized hydroxyapatite. In contrast, agarose gels allowed for uniform distribution of the particles. (Figure 4A). Hypertrophic chondrocytes triggered by thyroid hormone (DZC + T3) and deep zone chondrocytes (DZC) were evaluated using hydroxyapatite-loaded scaffolds of both nano and micro sizes. After 14 days of implanting the agarose/nHAp composite with DZC + T3 cells, a significant increase in ALP activity was observed in comparison to the control agarose scaffold. Similarly, the addition of nHAp on day 14 significantly increased the production of collagen X and the expression of Indian Hedgehog (Ihh). (Figure 4B). The incorporation of nHAp into agarose gels increased their compression modulus. (Figure 4C). Moreover, a correlation was observed between the compressive modulus and collagen content in the nHAp scaffold when compared to the control and microHAp scaffolds. (Figure 4C). However, no significant influence on DZC response was observed in relation to the size of particle modification (Khanarian et al., 2012). Future scaffolds may integrate both particle sizes, as both nano crystals and micro aggregates are present in the border of nature tissue (Zizak et al., 2003).

## 6 Nanoengineered bone-tendon interface

Tendons serve the purpose of connecting muscles to bones and exhibit structural similarities to ligaments. The majority of tendon injuries or ruptures commonly manifest in joints such as the knee or shoulder, and the resulting damage can be severe enough to disrupt the interface between the tendon and bone. Traditionally, these injuries have been stabilized through surgical intervention, as previously indicated, which can potentially lead to further complications. The predominant techniques employed for tendon healing typically encompass the utilization of artificial tendon implants, as well as the implantation of allogenic or xenogeneic grafts. For instance, arthroscopic treatment of irreparable rotator cuff injuries is performed with irreparable rotator cuff tears (Bond et al., 2008), TissueMend (produced by processing FBS through chemical and mechanical processes) (James et al., 2010), Porcine small intestinal submucosa cell-free biomaterial (Zheng et al., 2005), PermacolTM (mixes the strength and durability of artificial surgical repair substances with the biocompatibility of natural components) (Harper, 2001), and Polypropylene mesh (Ueno et al., 2004). Due to the origin and the xenogeneity of these transplants, individuals may experience severe immunogenic effects.

Moreover, the elastic moduli of tendons that have been rejuvenated with these scaffolds are observed to be lower in comparison to those of the native tissue (Derwin et al., 2006). The bone-tendon interface consists of mineralized fibrocartilage on the bone side and nonmineralized fibrocartilage on the tendon side. The native tissue in this region exhibits a gradient structure, and recent scholarly investigations have been centered on replicating these stepped structures through the use of multiple cells or nanomaterials (Seidi et al., 2011). The organization of bone-tendon interfacial areas is commonly characterized by longitudinally aligned collagen fibers. These fibers are comprised of cells that are arranged within the matrix (Butler et al., 2008). This strategy was used in one study to produce a PLGA nanofibrous scaffold, and it was discovered that fiber alignment influences cell morphology; fibroblasts seeded on allied fibers were more evenly distributed, whereas those cultured on random fibers displayed an unusual polygonal shape (Moffat et al., 2009). The expression of a2, b1, and aV integrins by cultured rotator cuff fibroblasts provided supporting evidence for this claim. Furthermore, the elastic modulus of the symmetrically ordered fibers exhibited a higher value (0.34 GPa) compared to that of the freely oriented fibers. (0.107 GPa). *In vitro*, the erratically aligned fibers demonstrated an accelerated degradation profile. This study also demonstrates that the cells in these soft tissue regions are capable of recognizing fiber alignment and directing their proliferation. In addition to being more physiologically relevant than microfibers, nanofibers possess enhanced biomimetic capability (Moffat et al., 2009).

**FIGURE 6:**
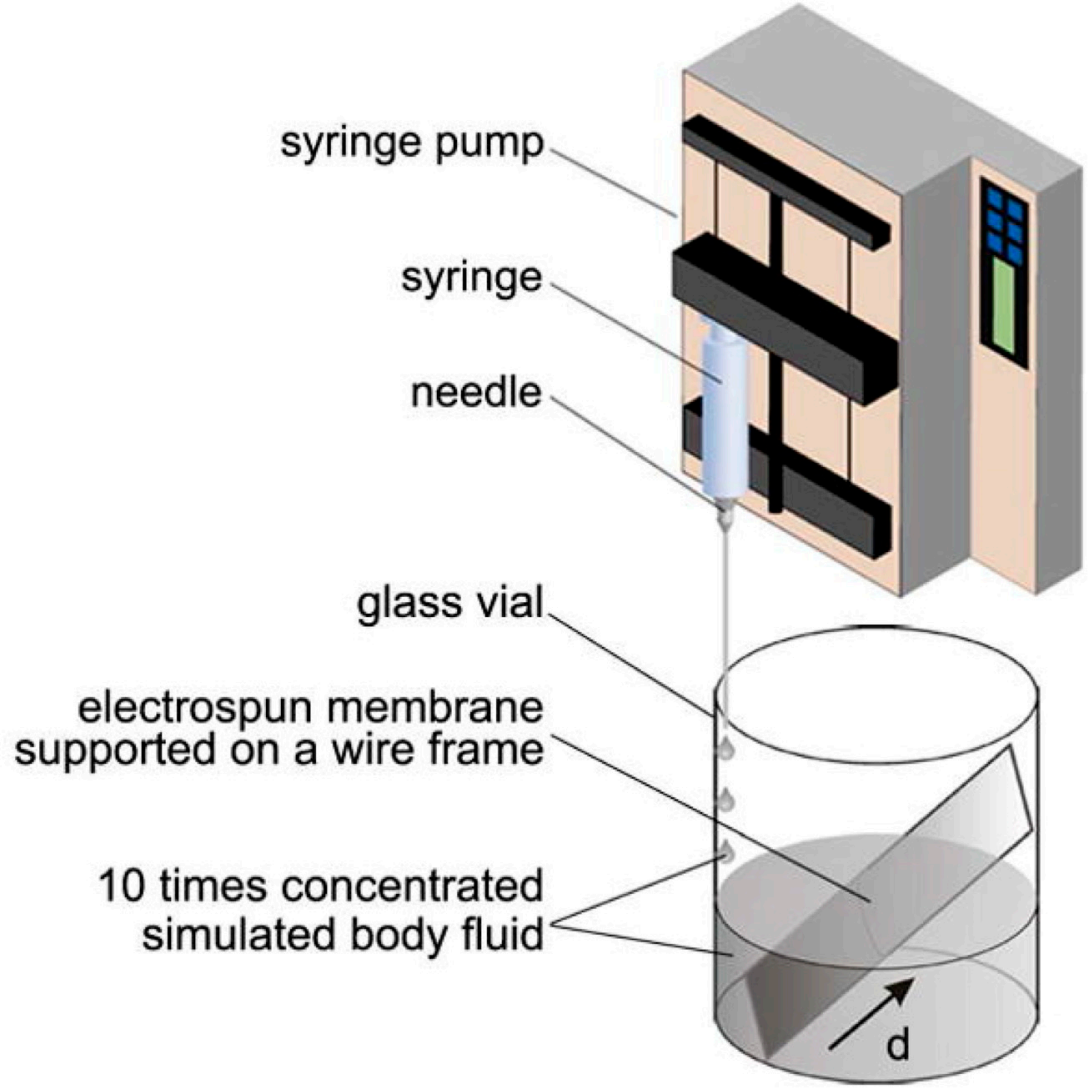
A diagram of the method for producing a graded calcium phosphate coating on an electrospun nanofiber nonwoven mat. To reduce accumulation time linearly from the bottom to the top of the substrate, tenfold focused simulation fluid was applied at a constant rate. The parameter d indicates the distance from the substrate’s lower edge (Li et al., 2009).

In addition, PLGA nanofibers have been designed with mineral gradients of hydroxyapatite to create a controlled environment for cell osteogenic development. One research (Liu et al., 2014) highlighted the utilization of fibrous scaffolds in conjunction with a mineralization sequence to promote osteogenesis. To accomplish this, rotator cuff fibroblasts may be co-cultured to generate a transitional environment for bone and tendon that is capable of regenerating the interfacial area. In another research study (Li et al., 2009), a mineralized graded scaffold was created using gelatin-coated PCL electrospun fibers and plasma-treated PLGA fibers (Figure 6). The choice of calcium phosphate as a coating was made to promote cellular division and proliferation. The MC3T3 cells demonstrated a positive correlation with increased calcium phosphate concentrations when cultured on the scaffolds. Additionally, the mechanical properties of the nanofibers were influenced by the gradient in mineral content observed throughout them. The mineral gradient resulted in a spatial gradient in the scaffold’s hardness, with higher mineral concentrations on the gradient leading to higher modulus values, suggesting a hardening effect on the nanofibers (Li et al., 2009). According to a recent study, it has been observed that calcium phosphates possess the potential to hinder the process of osteogenic differentiation due to their restricted crystallinity and elevated dissolution rate (Liu et al., 2014). The multilayered scaffold was constructed using various components, including a network of collagen crosslinks in the tendon area, a matrix consisting of collagen and chondroitin sulphate in the uncalcified fibrocartilage area, a relatively small amount of nHAp incorporated within the collagen matrix in the fibrocartilage calcification zone, and collagen with a high nHAp content in the bone region (Soo Kim et al., 2014). The introduction of nHAp resulted in different morphologies in the calcified fibrocartilage and bone areas, and the penetration of HA crystals into the collagen matrix decreased the pore size in the bone area. Furthermore, the mechanical properties of each stratum were found to be similar to those of normal tissue, in addition to the variation in pore size. An increase in the elastic modulus was observed at the interface between the tendon layer and the bone layer. Subsequently, human fibroblasts, osteoblasts, and chondrocytes were cultured on the stratified scaffold within the respective regions of the tendon, fibrocartilage, and bone. Using fluorescence imaging and SEM, uniform delivery of cells was detected on each layer (Soo Kim et al., 2014). The study presents a novel design methodology that has the potential to serve as a model for the development of natural interface tissues.

## 7 Drug delivery system based on nanotechnology

**FIGURE 7:**
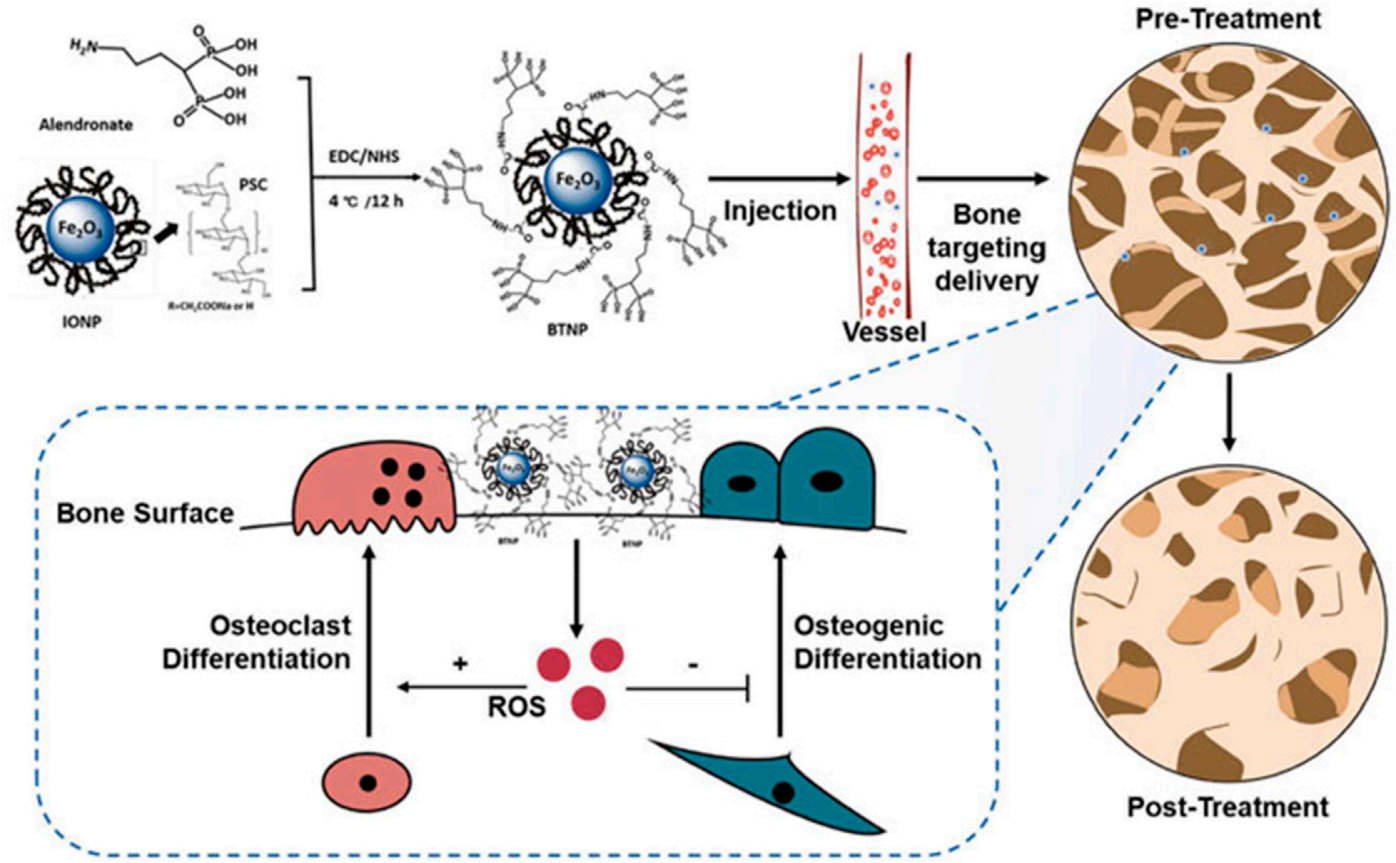
Alendronate and IONPs were used to make the BTNPs. The BTNPs were accurately transported to the bone tissues after being administered intravenously into osteoporotic mice. The osteogenic and osteoclast differentiation was regulated by adjusting the local ROS concentration, and OVX-induced osteoporosis was treated (Zheng et al., 2022).

Significant advancements have been observed in the field of nanotechnology-driven drug delivery systems (DDS) designed for the localized, sustained, and targeted administration of pharmaceutical agents. These advancements have demonstrated enhanced therapeutic efficacy and reduced the occurrence of adverse effects, rendering them particularly advantageous in the field of orthopedics. Numerous nanomaterials derived from nanotechnology with distinct chemical, physical, and biological properties have been used to develop innovative DDS for protecting, transporting, and precisely releasing pharmaceutical compounds. Traditional pharmaceuticals are limited by a lack of specific selectivity accompanied by severe side effects, the inability to traverse certain biological barriers, and inefficacy due to poor solubility (Lu et al., 2021). Nanotechnology-based DDS possess distinct advantages that allow them to overcome the limitations mentioned. These advantages include enhanced targeting capabilities, leading to reduced toxicity and increased bioavailability. Additionally, their large surface area to volume ratio facilitates effective drug introduction, while their nano-scale size enables them to cross biological barriers. Moreover, the abundance of surface chemistry in these systems enables interactions with biological targeting molecules (Yang et al., 2020). In the medical profession of orthopedics, nanotechnology-based DDS, such as metallic NPs, polymeric NPs, and lipid NPs, have contributed to innovation (Yang et al., 2019). These intelligent DDS have been widely employed in the identification and treatment of bone-related disorders, such as orthopedic oncology, osteoarthritis, orthopedic infections, osteoporosis, and cartilage/bone tissue regeneration. Their utilization aims to enhance the precision and efficacy of current therapeutic approaches. Osteoporosis (OP) is a prevalent progressive and deteriorating orthopaedic disease that affects millions of people. However, current anti-osteoporotic medications, such as calcitonin, bisphosphonates, and vitamin D, are systemically administered, resulting in adverse effects. Therefore, the development of novel DDS with enhanced treatment efficacy is both extremely desirable and a formidable challenge. Recent osteoporosis interventions are based on the direct control of bone metabolism (i.e., promoting bone resorption and inhibiting bone development) (Figure 7). Zheng et al. (Zheng et al., 2022), designed and synthesized a new bone-targeting antioxidative nano-iron oxide (BTNPs) in combination with alendronate, an Food and Drug Administration-approved clinical bisphosphonate. This technology may efficiently target the bone’s surface and favorably regulate the balance between bone resorption and bone formation *in vivo.* OA, a prevalent joint disorder with few therapeutic interventions, is also amenable to drug distribution *via* nanotechnology. The primary limitations of osteoarthritis treatment are rapid clearance following intra-articular injection and insufficient cartilage targeting following systemic administration. To overcome these limitations, extensive research has been conducted on DDS utilizing nanotechnology. According to the findings of Wei et al., the utilization of novel polymeric micellar NPs in conjunction with transforming growth factor (TGF) exhibited remarkable characteristics such as exceptional biocompatibility, stability, prolonged joint retention, and effective infiltration into cartilage. After the administration of intra-articular injection, the TGF-NPs demonstrated a high degree of effectiveness in mitigating the deterioration of OA cartilage, the sclerosis of the subchondral bone plate, and the post-surgical joint discomfort (Wei et al., 2021). Alternative approaches for drug distribution, such as Kartogenin, have been administered using recyclable poly(lactic-coglycolic acid) (PLGA). PLGA is a small chemical compound that can convert bone marrow-derived mesenchymal stem cells into chondrocytes. It has been demonstrated to facilitate rapid defect correction and the generation of hyaline cartilage (Shi et al., 2016). Nanotechnology-based DDS has attracted a great deal of attention for its potential to improve the treatment of bone-related disorders. Even though there have been significant advancements in the field of nanotechnology-based DDS for orthopedics, there are still concerns regarding the long-term safety and metabolic pathways.

**FIGURE 8:**
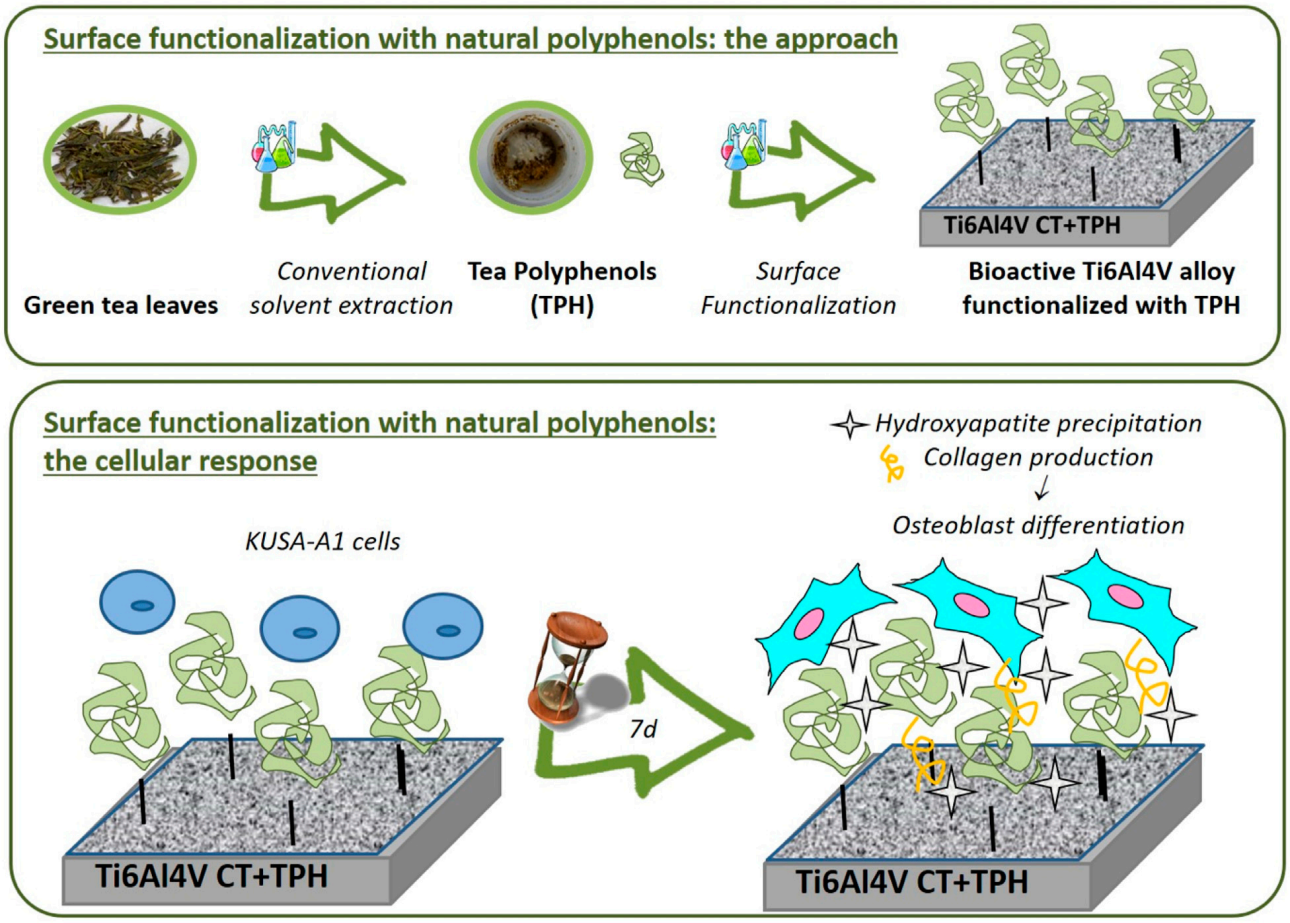
Chemically-treated titanium alloy (Ti6Al4V) surface. Green Tea Polyphenols in Combination with a Bioactive Titanium Alloy Surface (Cazzola et al., 2018).

Drug delivery from metal surfaces plays an important role in orthopedic applications as well. Using an extract of green tea polyphenols (tea polyphenols, TPH), the surface of a Ti alloy (Ti6Al4V) was functionalized to promote the precipitation of HA from body fluids (inorganic mineralization activity). Using a laser microscope, a greater quantity of extracellular matrix was observed on operational specimens, and fluorescence images revealed that these specimens contained more viable cells and osteocalcin. These findings demonstrate the ability of polyphenols to promote cell differentiation and biological mineralization, indicating that surface functionalization of graft metals may be a viable strategy for enhancing osteointegrability (Figure 8) (Jäger et al., 2022).

## 8 Nanotechnology-based orthopedic oncology diagnosis

NPs can incorporate ligands that selectively bind to specific molecules present on the surfaces of target cells. This enables the NPs to attach to the cells, thereby enhancing their biocompatibility and facilitating their integration into the host tissue microenvironment with greater efficiency. By the incorporation of contrast agents into ligand complexes, precise imaging of tumors at the cellular level can be attained (Susa et al., 2011). In addition, NPs containing contrast agents can improve the accuracy of targeted cancer imaging and evaluate tumor viability, which may be useful for perioperative evaluation and operational planning (Gavaskar et al., 2018). Cai et al. (2006), demonstrated that arginine-glycine-aspartic acid combined with NPs can be employed in the future to find the target malignancy and perform imaging-guided operations. The targeted tissue will be subjected to imaging using NPs capable of selectively detecting internal structures within viable cancer cells through the use of conventional fluorescence probes. Consider Simon’s research (Hennig et al., 2015), in which they devised a novel intracellular staining technique that carefully manages the tagging operation and allows direct access to the internal structure of live cells. The operational fluorescent probe was introduced into cells through the application of electrophoretic force using a porous glass capillary with a diameter of 100 nm. In the context of fluorescence microscopy staining, it is possible to promptly modify and observe the labeling density. To demonstrate the potential of this intracellular labeling method, a selection of commercially available cell permeable and impermeable fluorescent probes were applied to cells and imaged. Utilizing nanotechnology can revolutionize the early diagnosis of orthopedic tumors.

Standard orthopedic oncology treatment has inherent problems, such as drug resistance, toxicity to healthy host cells at the systemic level, and poor performance in providing excellent components that target cells, whereas nanotechnology-based orthopedic oncology diagnosis and treatment techniques have the potential to solve these issues (Zhang et al., 2019). NPs can transport substantial drug concentrations through fluids and blood in a secure manner. Specialized tumor cell-expressed ligands with enhanced NPs-binding properties can facilitate drug release at tumor sites. The ability to target antitumor drugs *via* specific ligands produced by tumor cells has the potential to enhance treatment parameters for primary bone cancers. Tumor cells possess surface proteins characterized by multidrug resistance, which effectively diminish the intracellular concentration of pharmaceutical compounds by actively transporting them out of the cells. Nanotechnology has the potential to not only effectively transport anticancer drugs to cancer cells, but also facilitate the delivery of specific genes aimed at countering proteins associated with multidrug resistance. Due to their diminutive size (passive targeting) and their ability to permeate cancer cells, NPs also facilitate increased drug concentrations within tumor cells. The prevention of osteogenic malignancies can be achieved through the downregulation of specific target genes and the use of specific NPs. Certain fusion oncogenes and molecular markers associated with osteogenic tumors can have their expression downregulated by targeted NPs. In addition to this, a considerable number of patients diagnosed with bone tumors necessitate orthopedic implants due to extensive bone resection. Nevertheless, it is worth noting that only a limited range of conventional implant materials are specifically designed to facilitate proper bone growth or mitigate the risk of tumor recurrence. Nanotechnology advancements have revealed an optimistic possible context. Nanomaterials are widely regarded as highly suitable for addressing substantial bone deformities (Luthringer et al., 2013). Phong et al. (Tran et al., 2010), showed that selenium nanoclusters produced on Ti surfaces can effectively inhibit the progression of bone cancer, while concurrently promoting the growth of healthy bone tissue. Some material properties can also promote bone growth and reduce osteolysis on the prosthetic surface (Puckett et al., 2010).

## 9 Challenges of nanotechnology commercialization

There are many obstacles to the development of nanomedicine, but one of the most significant is the pharmaceutical and medical device industries’ still-modest interest in this new technology. Currently, entrepreneurs are actively exploring numerous nanotechnology-driven proposals aimed at enhancing the diagnosis and treatment of illnesses. However, they are encountering challenges in securing partnerships with prominent pharmaceutical or healthcare device companies that are willing to license their technology or engage in collaborative efforts to obtain regulatory approval for their novel nanomedicine approaches. This issue is not entirely new to the medical industry, as it recalls the development of biotechnology-based medications over the last three decades. In addition to a lack of interest from large pharmaceutical companies, experts warn that there is a foundational reason for the slow pace of nanotechnology adoption in the healthcare industry, particularly in Europe: According to experts, the cost-regulated markets of major EU25 nations pose a substantial barrier to the development of innovative, high-value pharmaceuticals, including nanomedicine. Furthermore, the predominant therapeutic and diagnostic advantages of nanotechnology-based medications and contrast agents will primarily arise from their inherent ability to selectively target diseases with greater precision and yield more accurate diagnostic information. As a consequence, the restriction of patient groups leads to a reduction in the potential market for nanoparticle products. This, in turn, poses a significant challenge in terms of recovering costs associated with regulatory approval and may ultimately result in an economically unfavorable outcome for the development process.

## 10 Challenges and opportunities

The safety, health, and environmental implications associated with specific nanomaterials, along with their regulated status, are among the most significant concerns. The legal requirements for its oversight and further development will either have a positive or negative effect on the responsible application and adoption of new technologies. The presence of legislative constraints can either establish a framework for responsible adoption and decision-making regarding technology or impose unwarranted obstacles to innovation and technology utilization. The primary objective of regulations is to effectively identify and mitigate potential hazards, while simultaneously avoiding the unnecessary generation of excessive data, delays in processes, and increases in costs. In this regard, US agencies such as the Food and Drug Administration (FDA) and the Environmental Protection Agency (EPA) have evaluated NPs for environmental sustainability throughout their life cycle, as well as the difficulty of obtaining and interpreting scientific data to establish the safety of manufactured nanomaterials, over the past two decades. In addition, international governance institutions are being established to address the benefits of nanotechnologies and mitigate their potential risks to human health and the environment. These institutions utilize various voluntary, standard-setting, regulatory, statutory, and other governance platforms.

Nanotechnology has only recently been used in orthopedic research, diagnostics, and treatment. Nanotechnology has changed the science and practice of orthopedic treatment in the brief period that it has been studied and implemented. Nanotechnology offers potential advantages in terms of infection rates, reoperation necessity, and the enhancement of bone development, thereby presenting a theoretically safer approach to medical interventions in the human body. In addition to expanding research on current nanotechnology methodologies, benefits, and risks, it is imperative to thoroughly analyze and address the regulatory, production, and pricing challenges. The manufacturing process of nanotechnology products poses challenges due to their inherent characteristics and intricate nature. These items may be inaccessible due to their high cost, and the current regulatory systems can be time-consuming, thereby delaying the implementation of research. If these issues are resolved, nanomaterials will become more accessible and their use in orthopedics will be promoted more effectively.

In the future years, orthopedic surgery might be transformed by nanotechnology. Future research directions in this field include:

1 Nanotechnology has the potential to provide personalized therapies for musculoskeletal ailments. By customizing patient care, it is possible to enhance outcomes and decrease complications.
2 Nanotechnology has the potential to be utilized in the advancement of novel methods for tissue rejuvenation and renewal. This may entail the application of growth factors and nanofibrous scaffolds to induce the proliferation of new tissue.
3 Nanotechnology has the potential to be utilized in the development of materials that possess properties comparable to those of real bone tissue. This could potentially lead to the development of biocompatible grafts that exhibit improved mechanical properties.
4 Intelligent Implants: Nanotechnology can be employed to create grafts that react to their surroundings. This might comprise using sensors to monitor graft performance or DDS that respond to variations in the body.

## 11 Conclusion and future prospective

Nanotechnology is promising for regulating the chemistry and topography of bioimplant surfaces to (i) realize biological relations and (ii) make a new implant nanosurface that recapitulates organ or tissue interactions. Biomaterials based on nanotechnology have the potential to offer advantageous properties for osteoblast functions, tissue regeneration, and bone development, thereby enhancing their function in orthopedic implants. Nanomaterials implants can be utilized in a variety of ways using functional nanocoatings or nanostructured surfaces on the implant surface. The future of nanophase biomaterials is dependent on the development of enhanced design techniques that can combine the advantages of nanomaterials with modern manufacturing processes. Prior to the clinical/commercial application of nanotechnology-based orthopedic grafts, it is crucial to investigate the potential health risks associated with cell-nanophase biomaterial interactions. In the near future, nanotechnology holds great promise for the development of innovative orthopedic implants in the field of medicine.

Hence, the utilization of nanotechnology in the development of conventional implants with specific attributes is deemed more favorable compared to the utilization of nanoparticle-based transplants. This effectively prevents the potential for nanomaterials to disperse and impact the toxicity of tissues. Given the aforementioned concerns, the implementation of regulation has been proposed as imperative. The development of nanostructured implants and prostheses is met with caution by companies, primarily due to the lack of established therapeutic advantages, potential toxicity concerns, and the associated high costs. Despite being in its early stages of development, nanotechnology holds significant promise in improving orthopedic diagnosis, management, and research. The performance of the commercial and service sectors validates the idea that nanotechnology will play a crucial role in the future of treatment. Additional research is required to fully comprehend the safety and usefulness of this innovative technology. Utilizing nanomaterials and other fabrication technologies, future research will almost undoubtedly concentrate on enhancing design techniques. It is essential to understand the molecular processes that underlie cell–nanobiomaterial interactions. Additionally, care must be taken when validating the biosafety of nanomaterials and mitigating their effects.

Current research focuses on the development of novel materials and strategies for surface modification. The development of novel coating techniques and strategies that more closely resemble the structure of human bone would result in a new generation of orthopedic implants with enhanced integration and bone regeneration. The recent development and use of 3D printing technology are becoming increasingly valuable to the field of orthopedics; however, the field of orthopedic implants has not been thoroughly investigated. Polymers are widely employed in the fabrication of 3D-printed bone replacements due to their suitability as filaments for fused deposition modeling, compatibility with stereolithography equipment, and applicability as mediums for direct ink writing. To reduce implant costs, maintain patient safety, optimize surgical techniques, and reduce the risk of infection, materials used in orthopedics will continue to evolve.
